# Rapid Development of an Effective Newcastle Disease Virus Vaccine Candidate by Attenuation of a Genotype VII Velogenic Isolate Using a Simple Infectious Cloning System

**DOI:** 10.3389/fvets.2020.00648

**Published:** 2020-09-17

**Authors:** Nannan Wang, Mei Huang, To Sing Fung, Qiong Luo, Jun Xian Ye, Qian Ru Du, Liang Hai Wen, Ding Xiang Liu, Rui Ai Chen

**Affiliations:** ^1^South China Agricultural University, College of Veterinary Medicine, Guangzhou, China; ^2^Zhaoqing Institute of Biotechnology Co., Ltd., Zhaoqing, China; ^3^Guangdong Province Key Laboratory Microbial Signals & Disease Control, Integrative Microbiology Research Centre, South China Agricultural University, Guangzhou, China; ^4^Zhaoqing Branch Center of Guangdong Laboratory for Lingnan Modern Agricultural Science and Technology, Zhaoqing, China

**Keywords:** newcastle disease, reverse genetics, attenuated strains, immune protection, vaccination

## Abstract

Genotype-matched vaccines provide ideal protection against infection caused by new Newcastle disease virus (NDV) genotypes or variants even in the vaccinated chickens. In this study, we report a protocol for attenuation and rapid development of a velogenic NDV isolate as an effective vaccine candidate, using a simple and reliable infectious cloning platform. Based on DHN3, a genotype VII velogenic NDV isolate, recombinant rDHN3 was rescued by co-transfection of plasmids expressing the genomic RNA, NDV proteins NP, P and L, and the T7 polymerase without using a helper virus. Subsequently, an attenuated strain rDHN3-mF was produced by substitution of residues from amino acids 112 to 117 in the DHN3 F protein with the corresponding sequence from the LaSota strain. Both rDHN3 and rDHN3-mF are genetically stable during propagation in cell culture and chicken embryos. Further characterization through determination of EID_50_, MDT and clinical assessments confirmed that rDHN3 is velogenic and rDHN3-mF lentogenic. Vaccination of one-week-old SPF chicks with inactivated rDHN3-mF produced much higher anti-DHN3 antibody response and better protection against live DHN3 challenge than did the commercial LaSota vaccine, providing 100% protection and much earlier viral clearance. This attenuated NDV isolate would merit further development into a vaccine product.

## Introduction

Newcastle disease (ND) is a highly contagious infectious disease of poultry caused by Newcastle disease virus (NDV). NDV is an enveloped, single-stranded, negative-sense RNA virus, belonging to the *Avulavirus* genus of the family *Paramyxoviridae*. The complementary NDV genome contains six open reading frames (ORFs) coding for 4 structural proteins, nucleoprotein (NP), matrix protein (M), fusion glycoprotein (F) and hemagglutinin-neuraminidase (HN) glycoprotein, and two non-structural proteins, phosphoprotein (P) and large polymerase protein (L). Additionally, two non-structional proteins, known as V and W, are produced by RNA editing during the P gene transcription (1).

NDV attacks multiple organs of chickens, including the respiratory, nervous and digestive systems, with a lethal rate up to 100%. This virus is able to infect over 240 species of birds and spreads by direct contact from the infected to healthy birds (2). NDV virulence is determined by multiple genetic factors, involving different tissue/cell tropisms and specific immunoresponses induced by viral replication in the infected birds. The assessment of virulence is internationally conducted according to the intracerebral pathogenicity index (ICPI). The ICPI scores > 1.5 are considered velogenic, between 1.5 and 0.7 are mesogenic, and <0.7 are lentogenic (3). Also, the mean death time (MDT) scores <24 hours (h) are considered velogenic, between 24 and 96 h are mesogenic, and >96 h are lentogenic (4).

Since the initial outbreaks in 1926, NDV has caused four defined panzootics in the world, resulting in huge economic losses to the poultry industry. After the third panzootic, NDV vaccines were used worldwide, which had effectively suppressed the large-scale outbreak of ND globally. However, small-scale outbreaks and sporadic cases were repeatedly found among the vaccinated farms in South America and Asia countries, and more new strains with higher virulence are emerging (5–8). Up to now, more than a dosen of NDV genotypes have been identified in the class II group, but only one genotype in class I group is confirmed based on the similarity of F protein sequences (9). Class I NDV genomes are composed of 15,198 nucleotides (nt) and found typically in waterfowl and shorebirds worldwide (10–14), and class II stains have the genome size of 15,186 nt or 15,192 nt (15) and are typically found in the wild-birds and poultry species (12, 14, 16).

Entering the 1990s, more diverse genotypes and increased NDV infections have been reported from vaccinated farms, indicating that traditional NDV vaccines may not be sufficient to protect against the newly emerging viruses belonging to genetically distant groups (17–20). Prominently, genotype VII NDVs have gradually emerged as the main prevalent strains worldwide, implying the urgent requirement for a more efficient vaccine against this virulent genotype. As the Commission of the European Communities (CEC, 1993) has recommended against the use of a vaccine strain with an ICPI value more than 0.4, only the lentogenic strains can possibly be used as a vaccine (21). Newer vaccines have been developed by using the herpesvirus vectored and the LaSota strain-based recombinant viruses through reverse genetics and recombination technologies (17–22).

NDV HN protein is responsible for attachment of the virus to the host cell for viral entry and detachment of the virus from the cell for viral budding as well as removing sialic acid from progeny virus particles to prevent their self-aggregation (23). It therefore determines the tissue tropisms of NDV. The F protein mediates the fusion of the viral envelope with the host cell membrane (24). Both F and HN proteins are the main antigens that stimulate the production of protective antibodies against NDV infection (25). The motif (aa112-117) at the proteolytic cleavage site in the F protein is relevant to systemic replication and virulence (26–28). Residues at the cleavage site are generally 112R/K-R-Q-K/R-R-F117 in the velogenic group and 112G/E-K/R-Q-G/E-R-L117 in the lentogenic group (29–31).

In this study, we isolated and characterized DHN3, a new isolate of genotype VII NDV, and rescued the recombinant virus rDHN3 using a simple and reliable infectious cDNA cloning system. Based on this cDNA clone, an attenuated virus, rDHN3-mF, was constructed by substituting residues at the cleavage site of the F protein. Further characterization of the replication, pathogenicity, antigenicity, and stimulation of protective immunoresponse revealed that this mutant virus would be a suitable vaccine candidate.

## Materials and Methods

### Cells, SPF Chicken Embryos, SPF Chickens and Virus

BHK-21 cells were cultured in DMEM supplemented with 10% FBS and 1% streptomycin-penicillin and incubated at 37°C supplied with 5% CO_2_.

SPF chicken embryos (9–11 days old) and 1-week-old white Leghorn SPF chicks were obtained from the SPF Experimental Animal Center of Xinxing Dahua Agricultural, Poultry and Egg Co., Ltd., approved number: SCXK (Guangdong) 2018-0019. The SPF chickens were housed in the isolators of Zhaoqing Research Institute. Handling of the chicks was performed strictly following the experimental animal care guidelines and the experimental protocol approved by the Animal Welfare and Ethical Censor Committee at South China Agricultural University. Animals that did not die during the experiments were euthanized before execution.

The local NDV isolate, DHN3, was isolated from an infected chicken that had been vaccinated with an inactivated type II LaSota vaccine in a poultry farm in Guangdong, China in 2015. The viral seed was plaque-purified and propagated in BHK-21 cells for 1 generation and then in SPF chicken embryos for 10 generations.

### RT and PCR

First strand cDNA was synthesized by using a Reverse Transcriptase M-MLV kit (Takara) with the Random 6, Oligo (dT) or a gene specific primer, according to the manufacturer's instructions. The viral RNA was prepared from the allantoic fluid of infected embryos with Trizol 15596-026 (Invitrogen) according to the manufacturer's instructions. PCR amplifications were conducted by using the Premix Taq (Takara) or Prime STAR GXL DNA Polymerase (Takara) and the protocol varied with the primers and the length of the PCR products according to the manufacturer's instructions. All primers and sequence verifications were done in Sangon Biotech (Shanghai) Co. (Sangon Biotech).

### Plasmid Construction

Ten sub-genomic plasmids were constructed for identification of the DHN3 genomic sequence. Ten PCR fragments were produced by RT-PCR with the Premix Taq kit and the primers individually (Table 1), and inserted into pMD19T vector (Table 1). Plasmid pBR322-PNP (1-2617nt) was produced by multiple fragments recombination. The NP, MINI, and P (1893-2583nt) fragments were amplified by PCR with the high fidelity Prime STAR GXL DNA Polymerase (Takara), the primers and the corresponding templates as indicated in Table 2. These three fragments were then recombined with the *Hind* III/*Nhe* I linearized pBR322 vector in one reaction by using the ClonExpress Multis One Step Cloning Kit (Vazyme) according to the manufacturer's instruction. Plasmids pBR322-PDP (2564-7408nt) and pBR322-LPD3 (7381-15192nt) were constructed in the same way as pBR322-PNP using primers and templates shown in Table 2. The PDP was composed of P (2584-3080nt), PD1, PD2 and the PD3 (6261-7383nt) regions and LPD3 was composed of the PD3 (7384-8283nt) and L regions.

**Table 1:** Primers used for sequencing the DHN3 genome.

**Primer**	**Sequence 5^′^−3^′^**	**Fragment (bp)**
N-F	ACCAAACAGAGAATCCGTGAGGTA	1,591
N-R	TCAGTACCCCCAGTCAG	
MINI-F	GAACAAGCCGCAAGGG	536
MINI-R	CATCTGCAGACAGTCCCACTGGTCTCAAGTAT	
P-F	CATACTTGAGACCAGTGGGACTGTC	1,185
P-R	CAGGAGCCTGCTATGAGT	
PD1-F	TGGGAGTGGAGAAGGAC	2,022
PD1-R	GTTATCTGTGCCGCTGT	
PD2-F	GACTCCATTCGCAAGA	1,617
PD2-R	CATTCTCCAGCACGAC	
PD3-F	CCGATAACCTGTCAAGTAG	2,023
PD3-R	GTCAGCATTGTCGGATT	
L1-F	GGATGGTTGGGAGGACGAC	2,544
L1-R	GCAACTGCGTCAACACCA	
L2-F	GTTAGCAATGAGTCAACTGTCC	2,126
L2-R	CTTCTTCGGTGAACAGCCTTTG	
L3-F	CTACAGAGTGTCGCCTTAC	2,196
L3-R	GGTAAAGGGCTGGATAGGAGA	
L4-F	TCTCCAATGGCTATGCCTGTA	977
L4-R	GCGCACCAAACAGAGATTTGGTGAATGAC	

**Table 2:** Plasmids, templates, and primer sequences.

**Plasmid**	**Template**	**Primer and sequence 5^′^−3^′^**
pBR322-PNP	pMD19-N	C-NP-F: **CATCTGGTTGCCCTTGCGGCTTGTTC** C-Sac11BtNhe-ST-R: **ATTGCATCAACGCATATAGCGCTAGC** ***GCGATG***T ACCGCGGATCGTTACCAAACAGAGAATCCGTGAGGTAC
	pMD19-MINI	C-Mini-F: **GACAGTCCCACTGGTCTCAAGTATG** C-Mini-R: **GAACAAGCCGCAAGGGCAACCAGATG**
	pMD19-P	C-HindBt-P-F: TGTTTGACAGCTTATCATT **CGATAAGCTTCCTCCATCA** **TAGACAT*****CATCGC***CT C-P-R: **CATACTTGAGACCAGTGGGACTGTC**
pBR322-PDP	pMD19-P	C-P-F: **GACAGTGTCCTTCTCCACTCCCATG** C-BtNhe-P-R: **ATTGCATCAACGCATATAGCGCTAG** ***CAGGCGATG***AT GTCTATGATGGAGG
	pMD19-PD1	C-PD1-F: **GACCCTTGGATCTTGCGAATGGAGTC** C-PD1-R:**CATGGGAGTGGAGAAGGACACTGTC**
	pMD19-PD2	C-PD2-F: **GTCTCCTACTTGACAGGTTATCGG** C-PD2-R: **GACTCCATTCGCAAGATCCAAGGGTC**
	pMD19-PD3	C-HindBtg-PD3-F: TGTTTGACAGCTTATCATCGATAAG **CTTGT*****GCGAT*** ***G*TCACTGGGTGAATTAGG** C-PD3-R: **CCGATAACCTGTCAAGTAGGAGAC**
pBR322-LPD3	PMD19-PD3	C-PD3-F: **CGCAATGTCGTCCTCCCAACCATCC** C-BtNhe-PD3-R: ATTGCATCAACGCATATAGCGCTAGCCCTAA
		**TTC**
		**ACCCAGTGA*****CATCGC*****AC**
	pXJ40-L	P-L2-R: **GTGAATGTAAGGCGACACTCTGTAG** C-L1-R: **GGATGGTTGGGAGGACGACATTGCG** P-L3-F: **CTACAGAGTGTCGCCTTACATTCAC** C-HindBtNot-L4-F: TTGACAGCTTATCATCGATAAGCTT***GCGATG***
		AGCGGCCG
		CT**CCATTAATACGACTCACTATAGGACC**
		**AAACAGAGATTTGGTGAATGAC**

*The restriction enzyme cutting site is in italic; the homologous sequence is in bold*.

Construction of pBR322-Base: a DNA fragment containing the T7 promoter, T7 terminator, HDV (Hepatitis delta virus) Ribozyme, HC1 and HC2 sequences was synthesized from Sangon Biotech (Shanghai) Co (Sangon Biotech), and inserted into pBR322 vector between *Not* I and *Nhe* I sites.

Construction of pBR322-DHN3: pBR322-PNP was digested with *BtgZ* I/*Hind* III to release the PNP fragment; pBR322-PDP was digested with *BtgZ* I to release the PDP fragment; pBR322-LPD3 was digested with *BtgZ* I to release the LPD3 fragment. These three fragments were then ligated with T4 ligase (NEB) to produce the DHN3 full-length fragment A. The vector based fragment was produced by PCR amplification from pBR322-Base with primers pBR322-Base-F (5′-ATCGGTAGAAGGTTCCCTCAGGTTC-3′) and pBR322-Base-R (5′-GGTCCTATAGTGAGTCGTATTAATG-3′). The DHN3 fragment was then recombined into the vector fragment using the ClonExpress Multis Kit (Vazyme) and produced the pBR322-DHN3 plasmid, which was further verified by sequence verification (Sangon Biotech).

Three auxiliary plasmids were constructed by inserting DHN3 genes coding for NP, P and L proteins, respectively, into pXJ40 (Table 3), which has a strong CMV promoter and a start codon with the favorable Kozak context sequence for efficient gene translation. For construction of pXJ40-L, pXJ40 was linearized by *BamH* I /*Pst* I digestion. The L was composed of L1-L4 fragments generated by PCR with the specific primers and templates indicated in Table 3, and these three PCR fragments were then recombined with a *BamH* I/*Pst* I linearized pXJ40 vector by using the ClonExpress Multis Kit (Vazyme). For construction of pXJ40-NP, pXJ40 was linearized by *EcoR* I/*Xho* I digestion. The NP fragment was amplified by PCR with the primers and templates indicated in Table 3. This PCR fragment was digested with *EcoR* I/*Xho* I then ligated to the linearized pXJ40 vector by using the T4 DNA ligase. For construction of pXJ40-P, pXJ40 was linearized by *EcoR* I/*Xho* I digestion. The P fragment was amplified by PCR with primers and template indicated in Table 3. This PCR fragment was digested with *EcoR* I/*Xho* I then ligated to the linearized pXJ40 vector by using the T4 DNA ligase.

**Table 3:** Primers for construction of helper plasmids.

**Primer**	**Sequence 5^′^−3^′^**
pXJ40-NP-F	*GAATTC*GCCACCATGTCGTCTGTTTTTGACGAATACGAGC
pXJ40-NP-R	*CTCGA*GTCAGTACCCCCAGTCAGTGTCG
pXJ40-P-F	*GAATT***C**GCCACCATGGCTACCTTTACAGATGCGGAG
pXJ40-P-R	*CTCGA*GTCAACCATTCAGCGCAAGG
pXJ40-L1-F	**ACTCACTATAGGGCGAATTC*****GGATCC***GGATGGTTGGGAG
	GACGACATTG
pXJ40-L1-R	**GGACAGTTGACTCATTGCTAACATA**
pXJ40-L2-F	**TATGTTAGCAATGAGTCAACTGTCC**
pXJ40-L2–R	**GTGAATGTAAGGCGACACTCTGTAG**
pXJ40-L3-F	**CTACAGAGTGTCGCCTTACATTCAC**
pXJ40-L3–R	**CGAATATCAGGTAACACTCCATATC**
pXJ40-L4-F	**GATATGGAGTGTTACCTGATATTCG**
pXJ40-L4-R	**TAAGATCTGGTACCGAGCTC*****CTGCAG***GCGCACCAAACAG
	AGATTTGGT
pXJ40-DE3-F	**ACTCACTATAGGGCGAATTC***GGATC*CGCCATGAACACGA
	TTAACATCGC
pXJ40-DE3-R	**TAAGATCTGGTACCGAGCTC***CTGCAG*TTACGCGAACGCG
	AAGTCCGACTC

*The restriction enzyme cutting site is in italic and the homologous sequence is in bold*.

The DE3 gene capable of expressing the T7 RNA polymerase was amplified from BL21 bacterial DNA and inserted into pXJ40 via recombination to produce plasmid pXJ40-DE3 (Table 3). Briefly, pXJ40 was linearized by *Pst* I/*BamH* I digestion. The DE3 gene fragment was amplified from the genomic DNA extracted from BL21 bacteria by PCR with primers indicated in Table 3. This PCR fragment was then recombined into the *Pst* I/*BamH* I linearized pXJ40 vector by using the ClonExpress Multis Cloning Kit.

### Construction of the Full-Length DHN3 and DHN3-mF Clones

**Figure 1:**
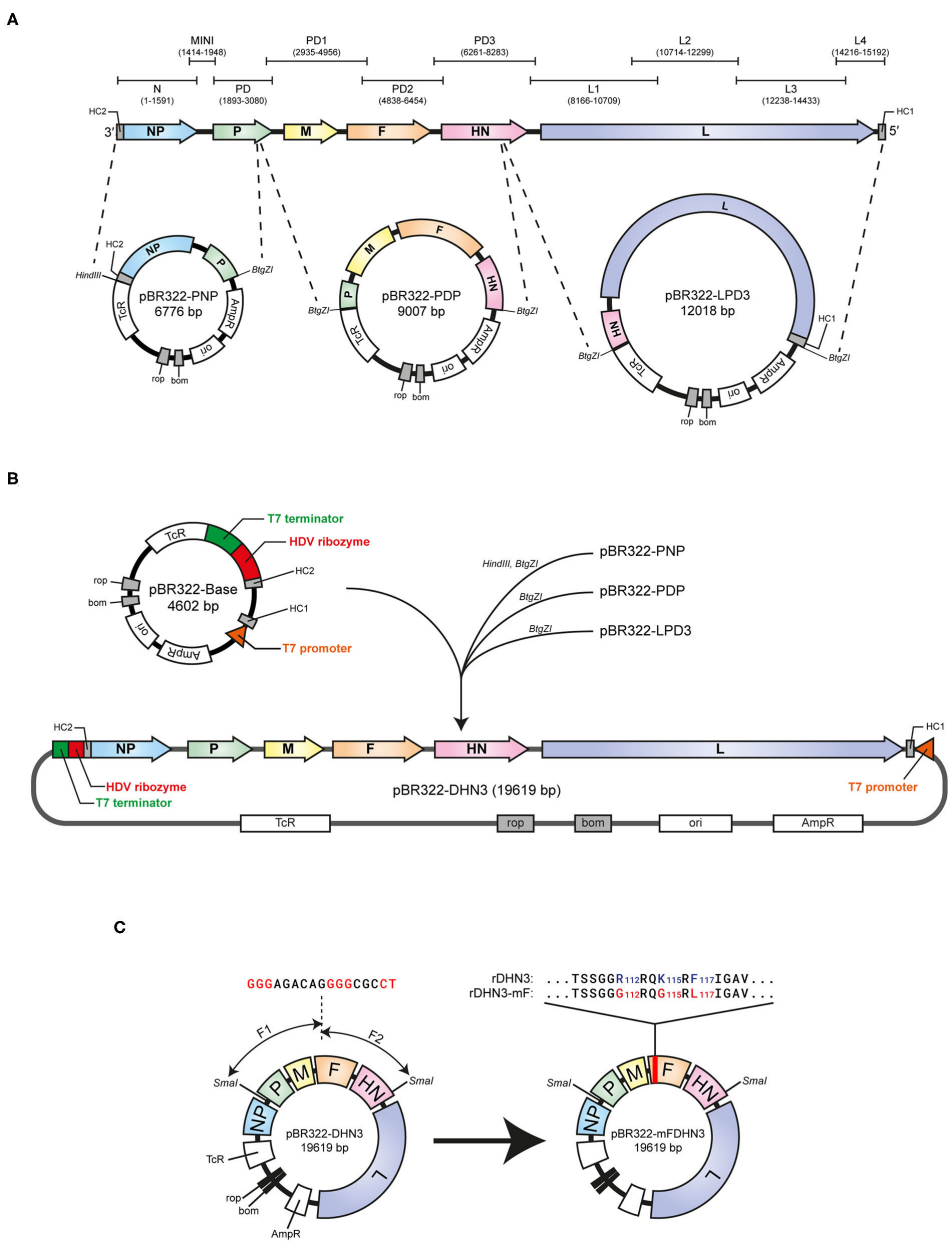
Genome organization of NDH3 and the schematic strategy for construction of the full-length pBR322-NHD3 and mutant pBR322-NHD3-mF constructs. **(A)** Ten overlapping PCR fragments covering the whole DHN3 genome are indicated by N, MIMI, PD, PD1, PD2, PD3, L1, L2, L3, and L4, respectively. The three subgenome-sized plasmids, pBR322-PNP, pBR322-PDP, and pBR322-LPD3, were constructed based on the indicated restriction sites and used for the construction of pBR322-DHN3. **(B)** The pBR322-Base plasmid was constructed by inserting a synthetic DNA fragment into pBR322 between Not I and Nhe I site. The DNA fragment is composed of the T7 terminator, HDV ribozyme, HC2 (5′ end from 1-141nt of DHN3), HC1 (3′ end from 15192-15159nt of DHN3) and the T7 promoter sequence. The PNP, PDP and LPD3 fragments excised from the three subgenome-sized plasmids were ligated, and then recombined with pBR322-Base to produce the full-length pBR322-DHN3. **(C)** pBR322-DHN3-mF was constructed by recombination of three fragments. The large fragment prepared from SmaI- digestion pBR322-DHN3, and the two smaller fragments containing the mutated sequence marked in red were generated by PCR amplification with two pairs of specially designed primers (Table 4).

To construct the full-length infectious clone based on DHN3, an artificial DNA fragment containing the T7 promoter, T7 terminator, HDV Ribozyme, HC1, and HC2 sequences shown in Figure 1B was synthesized through biosynthetic method. This DNA fragment was then inserted into pBR322 to produce the pBR322-Base plasmid, in which HC1 contains the 3′-end sequence of DHN3 from nucleotides15192-15159 and HC2 the 5′-end sequence from nucleotides1-141. The HC1 and HC2 sequences are able to provide two homologous arms for subsequent recombination with the rest of DHN3 genomic sequences.

The full-length DHN3 genomic sequence is divided into three fragments, PNP, PDP and LPD3, based on positions of the *BtgZ* I restriction sites indicated in Figure 1A. These three fragments were cloned into pBR322 by homologous recombination to produce pBR322-PNP, pBR322-PDP and pBR322-LPD3, respectively (Table 2). The PNP, PDP and LPD3 fragments from these plasmids by restriction enzyme digestion were joined together using the T4 DNA ligase to produce the full-length DHN3 fragment. Meanwhile, the pBR322-Base fragment (Figure 1B) was generated by PCR with pBR322-base-F/R primers. The full-length DHN3 fragment and the pBR322-Base fragment were then recombined by homologous recombination, producing pBR322-DHN3 (Figure 1B).

In order to attenuate the virulence of DHN3, three amino-acid-substitutions (R112-G, K115-G, and F117-L) at positions 112, 115 and 117 of the F protein, respectively, were introduced into the full-length DHN3 cDNA, resulting in the mutation of the furin-cleavage site at residues 112-117 from R-R-Q-K-R-F to G-R-Q-G-R-L, the same sequence as in the corresponding region of LaSota F protein. Through recombination of three fragments, this mutant was ideally constructed as shown in Figure 3B. The two DNA fragments between the two *Sma* I sites in the DHN3 genome were generated by PCR using two specifically designed primers (change-F1-F/change-F1-R and change-F2-F/change-F2-R) as indicated in Table 4. Recombination of these two fragments with the large fragment released from the *Sma* I digested pBR322-DHN3 produced pBR322-mFDHN3 (Figure 1C).

**Table 4:** Primers for construction of pBR322-DHN3-mF.

**Primer**	**Sequence 5^′^−3^′^**
F1-F	**CACCCCCAGCCACACGACCCCATCCA***CCCGGG*ACAACA
	CAGGCACAGCTCGGCCAG
F1-R	**CTATAAGGCGCCCCTGTCTCCCTC**CTCCGGACGTGGATAC
	AGACCCTTG
F2-F	**GAGGGAGACAGGGGCGCCTTATAG**GTGCTGTTATTGGCAG
	TGTAGC
F2-R	**CTTGGTGTTGCTTGAACTCA***CCCGGG*TGACACGACTGCGA
	GATATGTTG

*The restriction enzyme cutting site is in italic; the homologous sequence is in bold*.

### Virus Rescue

BHK-21 cells were co-transfected with pBR322-DHN3 or pBR322-mFDHN3, the three auxiliary plasmids (pXJ40-NP, pXJ40-P, pXJ40-L) and the T7 polymerase-coding plasmid pXJ40-DE3, using the LTX transfection kit (Thermo Fisher). After incubation for 4 h, the transfected cells were washed twice with PBS and incubated in DMEM supplemented with 1% FBS. For cells transfected with rDHN3-mF, 0.1% trypsin was also added to the culture medium. After incubation for 4 to 6 days, the cells together with the culture media were harvested and frozen at −80°C.

### Preparation of the Viral Stock

Viral stocks were prepared by centrifugation of the infected cells at 14,000 for 10 min at 4°C after 3 freezing and thawing cycles, and the supernatant was collected. Viral stock prepared from chicken embryos were prepared by infecting the chicken embryos with 0.1 mL of viral stock. The embryos were checked three times every day up to 3 days. The dead embryos were transferred to a 4°C refrigerator for 4–12 h, and the allantoic fluid was collected afterwards.

### The Viral Growth Curve

BHK-21 cells grown in 35 mm plates were infected with DHN3, rDHN3 or rDHN3-mF at an MOI of ~1 after washing twice with PBS. After attachment at 37°C for 2 h, cells were washed three times with PBS, then incubated in 2 mL of DMEM supplemented with 1% FBS. For cells infected with rDHN3-mF, 0.1% trypsin was supplemented. Total cells and culture media were collected at 4, 8, 16, 24, 36, 48, and 60 h post-infection, respectively, and kept at −80°C.

### Vaccination and Sample Collections

Forty-eight one-week-old SPF chicks were divided into 4 groups, 12 in each and housed in 4 isolators. Chicks were immunized with 10^7^EID_50_ dose each of inactivated rDHN3-mF, LaSota vaccine or sterile saline in 0.2 mL by the neck subcutaneous injection, and blood samples from each group were collected on 7, 14, and 21 days post-immunization for HI test. On 21 days post-immunization, all birds were infected with 10^5^EID_50_ of the virulent DHN3. Trachea and cloaca swabs were collected on 3, 5, and 7 days post-challenge.

### Viral Shedding Test

The swabs were re-suspended and mixed in 1 mL sterile saline supplemented with penicillin to the final concentration of 10,000 units/mL, and the mixture was stored at 4°C overnight. The mixture was centrifuged at 2,000 for 10 min at 4°C, and the supernatant from each sample was used to infect a group of three SPF chicken embryos (9–11 days old) at a dose of 100 μL per embryo. After incubation at 37°C for 48 h, the allantoic fluid was harvested from each embryo for HA test. If one out of three allantoic fluid samples in each group was HA positive, it was determined as active NDV shedding.

### HA, HI, and MDT Tests

HA and HI assays were performed using the standard microtiter plate method as recommended by the OIE [World Animal Health Information Database, 2018].

For MDT, the allantoic fluid was serially diluted with sterile saline to 10^−5^ ~ 10^−10^ and each 100 μL of the dilution was inoculated to five SPF chicken embryos (9–10 days-old). The embryos were checked three times per day for 7 days. Any embryo found to be dead within the first 24 h would be discarded. The Reed-Muench method was used to calculate the MDT of chicken embryos.

### Statistical Analysis

The one-way ANOVA method was used to analyze the significant difference between the indicated sample and the respective control sample. Significance levels were presented by the *p*-value (ns, non-significant; ^*^p < 0.05; ^**^p < 0.01; ^***^p < 0.001; ^****^p < 0.0001).

## Results

### Sequencing and Phylogenetic Analysis of DHN3, a Local NDV Isolate Classified as Genotype VII

**Figure 2:**
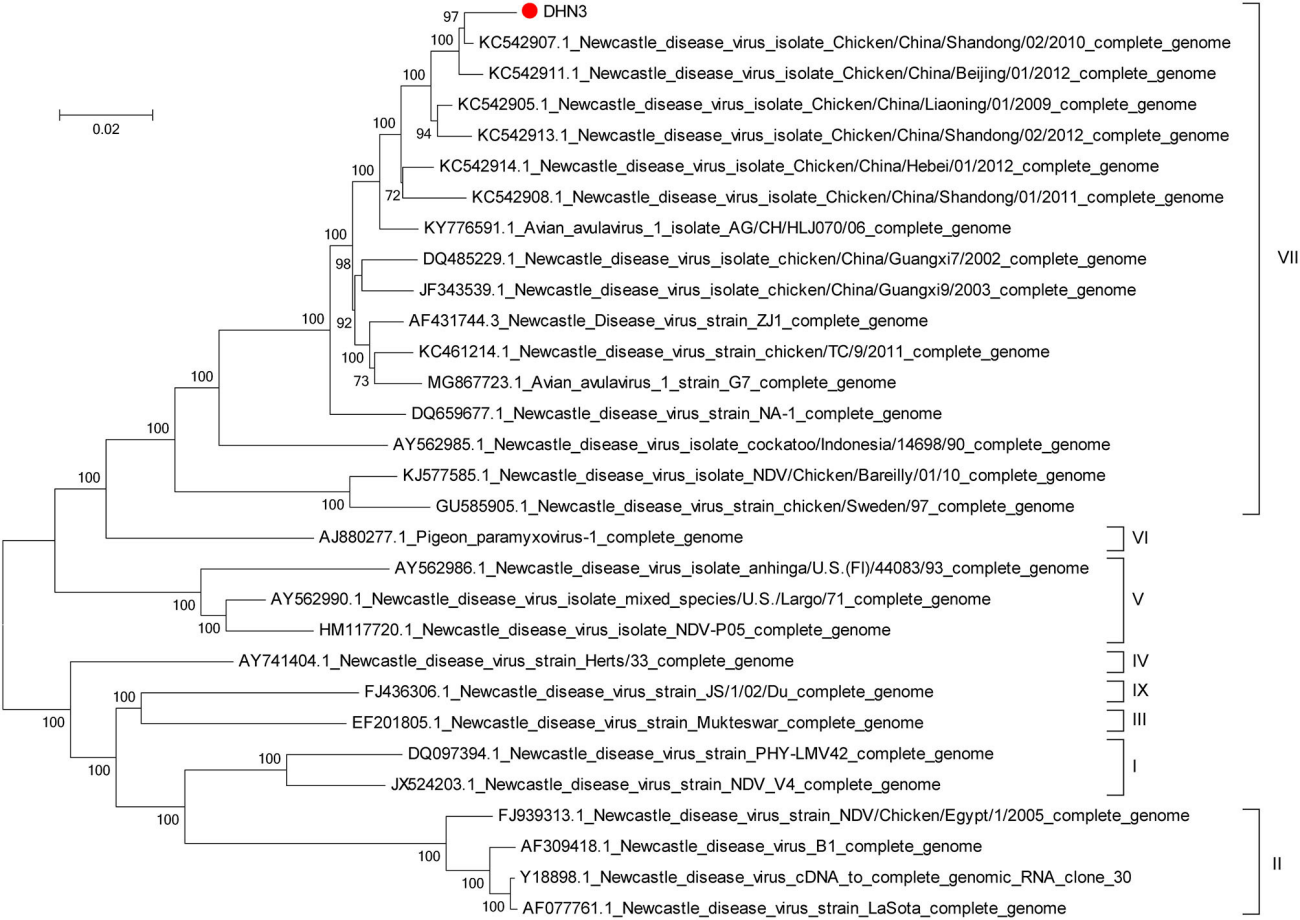
Phylogenetic analysis of DHN3. The phylogenetic tree of the complete DHN3 genomic sequence together with 29 published NDV sequences obtained from GenBank was constructed using the Maga6 software. The DHN3 isolate from this study is marked with a red dot.

The genome of DHN3 was sequenced from ten cDNA fragments produced by RT-PCR with specific NDV primers (Table 1), in which the primer sequences were conserved in NDV genomes available from GenBank. The ten PCR fragments were cloned into pMD19T-vector, yielding pMD19-N, pMD19-P, pMD19-MINI, pMD19-PD1, pMD19-PD2, pMD19-PD3, pMD19-L1, pMD19-L2, pMD19-L3, and pMD19-L4 (Figure 1A). The sequences of DHN3 in the ten plasmids were assembled and submitted to GenBank under the accession number MT447874. The phylogenetic relationship among 29 isolates of published full-length NDV sequences obtained from GenBank together with DHN3 was analyzed with the Mega6 software. The phylogenetic tree shown in Figure 2 demonstrates that DHN3 belongs to type VII.

### Rescue of the Recombinant Viruses rDHN3 and rDHN3-mF, and Characterization of Their Genetic Stability and Growth Kinetics in Cells

**Figure 3:**
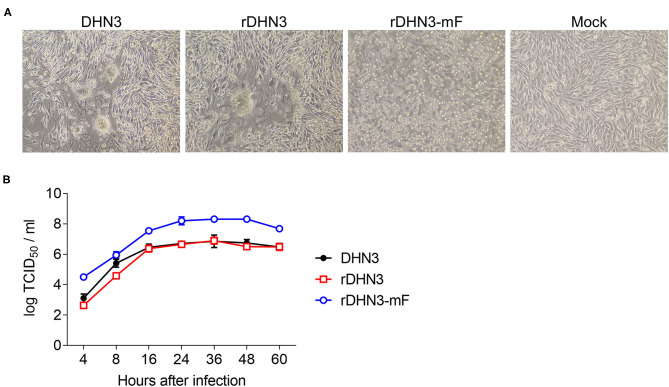
Growth kinetics of DHN3, rDHN3 and rDHN3-mF. **(A)** CPE formation. BHK-21 cells were infected with DHN3, rDHN3, rDHN3-mF, or mock-treated for 24 h. **(B)** Growth curves. BHK-21 cells were infected with DHN3, rDHN3 or rDHN3-mF at an MOI of 1, and harvested at the indicated time points. Viral titers were determined by TCID_50_.

The recombinant virus, rDHN3, was rescued from BHK-21 cells by co-transfection of pBR322-DHN3, the three ancillary plasmids (pXJ40-NP, pXJ40-P, and pXJ40-L) and pXJ40-DE3 as described in materials and methods. The cytopathic effect (CPE) of rDHN3 appeared to be similar to the original DHN3 and both showed the formation of syncytial cells (Figure 3A).

To rescue the recombinant virus (rDHN3-mF) carrying point mutations in the furin-cleavage site on the F protein of rDHN3, BHK-21 cells were co-transfected with pBR322-DHN3-mF, the three ancillary plasmids (pXJ40-NP, pXJ40-P and pXJ40-L) and pXJ40-DE3 in the presence of 0.1% trypsin. Differing from rDHN3, syncytium formation was not obvious in cells infected with rDHN3-mF, instead, extensive cell death was observed (Figure 3A).

The genetic stability of rDHN3 and rDHN3-mF was checked by verification of the F gene sequence from the 10th passage of rDHN3 and rDHN3-mF in SPF chicken embryos, respectively, confirming no reversion or other mutations.

The growth kinetics of rDHN3-mF, rDHN3, and the original DHN3 isolate were determined in BHK-21, revealing similar replication kinetics among them (Figure 3B). However, compared to rDHN3 and DHN3, about 10-fold higher titers of rDHN3-mF were produced at each time point. Statistical analysis confirmed that significantly higher titers of rDHN3-mF were produced, compared with either rDHN3 (*p* < 0.001 at all time points) or DHN3 (*p* < 0.001 at all time points except at 8 hpi *p* = 0.00313). No significant difference in the titers of rDHN3 and DHN3 was observed at all time point. These results demonstrate more efficient replication of rDHN3-mF in this cell line.

### Characterization of the Growth Kinetics and Virulence of rDHN3 and rDHN3-mF in Chicken Embryos

The growth kinetics of rDHN3-mF and rDHN3 in chicken embryos were analyzed by HA assay. The titers of both DHN3 and rDHN3 were 256, while rDHN3-mF was 512, higher than the two wild type strains (Table 5). Repeated experiments with different viral stocks were conducted and the same result was obtained. However, TCID_50_ determination indicated that there were no obvious difference among these three strains (Table 5). The TCID_50_ values for DHN3, rDHN3 and rDHN3-mF were 10^8.3^/mL, 10^9^/mL and 10^8.5^/mL, respectively.

**Table 5:** Pathogenic index of DHN3, rDHN3, and rDGN3-mF.

**Virus strain**	**HA**	**TCID_**50**_/mL**	**EID_**50**_/mL**	**MDT (h)**
DHN3	2^8^	10^8.3^	10^7.6^	46
rDHN3	2^8^	10^9.0^	10^7.5^	48
rDHN3-mF	2^9^	10^8.5^	10^8.7^	110

*The HA titer value is expressed as mean titer value ± standard deviation, n = 3*.

The virulence of DHN3, rDHN3, and rDHN3-mF were further studied by determination of EID_50_ (50% Egg Infectious Dose) and MDT. As shown in Table 5, the results demonstrated that a higher EID_50_ value and longer MDT were detected in the allantoic fluid collected from rDHN-mF-infected eggs compared to those from rDHN3-infected eggs. The EID_50_ value for rDHN3-mF was 10^8.7^EID_50_/mL, higher than the 10^7.6^EID_50_/mL value for DHN3 and 10^7.5^ EID_50_/mL for rDHN3; MDT was 110 h for rDHN3-mF, longer than the 46 h for DHN3 and 48 h for rDHN3 (Table 5). According to the category standard, DHN3 and rDHN3 belong to velogenic group, and rDHM3-mF lentogenic group.

### Assessment of the Virulence and Induction of Immunoresponse in Chicks Infected With DHN3, rDHN3 and rDHN-mF

One-week old SPF chicks (14/each group) were infected with DHN3, rDHN3, rDHN-mF, and LaSota, respectively, and sterile saline was used for mock infection control. The chicks were inoculated with 10^6^EID_50_/mL of each virus via the nasal and eye routes. The clinical manifestations were examined every day for 21 days. For the postmorten examination, three chicks in each group were chosen randomly on 3 and 7 days post-infection (dpi), and serum samples were collected on 7, 14, and 21 dpi. Generally, chicks infected with DHN3 or rDHN3 appeared drowsy, depressive, and discharging yellow-greenish thin stools starting from 2 dpi. Death was observed from 3 dpi, and by 4 dpi all birds were dead. However, chicks infected with rDHN-mF, LaSota or mock-infected appeared normal with good spirit, supple feathers and strong appetite throughout. Postmortem examination showed no obvious abnormality in the liver, spleen, trachea, stomach and duodenum from chicks infected with rDHN3-mF or mock-infected, but few individual chicks infected with LaSota showed swollen thymus and liver, and congested brain and heart. More severely enlarged liver and congested kidney, thymus and brain were observed in chicks infected with rDHN3 and DHN3.

HI tests were further performed to determine the immunoresponse induced in chicks infected with rDHN3-mF and LaSota, and antibody titers against DHN3 and LaSota were determined from the infected chicks on 7, 14, and 21 dpi, respectively. The results shown in Table 6a demonstrated that antibodies against either DHN3 or LaSota increased over time. In sera collected from chicks infected with rDHN3-mF, anti-DHN3 antibody titers increased from 51 on 7 dpi to 128 on 21 dpi, but the anti-LaSota antibody titers increased from 4 on 7 dpi to 20 on 21 dpi. In sera collected from chicks infected with LaSota, the anti-DHN3 and anti-LaSota antibody titers increased from 20 on 7 dpi to 51 on 21dpi, and from 13 on 7 dpi to 40 on 21 dpi, respectively. Chicks in the control group didn't show specific antibody against either DHN3 or LaSota.

**Table 6a:** Antibody response against DHN3 and LaSota.

**Group**	**Anti-DHN3 HI titer**			**Anti-LaSota HI titer**		
	**7**	**14**	**21**	**7**	**14**	**21 dpi**
rDHN3-mF	50.91 ± 1.49	64 ± 2	128 ± 2	4 ± 1	16 ± 2	20.11 ± 1.49
LaSota	20.11 ± 1.49	12.73 ± 1.49	50.91 ± 1.49	12.73 ± 2.22	40.22 ± 1.49	40.22 ± 1.49
Control	2.83 ± 1.64	2 ± 1	4 ± 1	2 ± 1	1	2 ± 1

*The HI titer is presented as the mean titer ± standard deviation, n = 3*.

Statistical analysis confirmed that infection of chicks with rDHN3-mF produced significantly higher antibody response against DHN3, compared to chicks infected with LaSota at all time points (Table 6b, rDHN3-mF vs. LaSota). Similarly, infection of chicks with LaSota also produced significantly higher antibody response against LaSota, compared to chicks infected with rDHN3-mF (Table 6b, LaSota vs. rDHN3-mF). However, significantly higher antibody response against DHN3 was induced in rDHN3-mF-infected chicks, compared to anti-LaSota antibody response in chicks infected with LaSota (Table 6b, Anti-DHN3 vs. Anti-LaSota). These results demonstrate that DHN3-mF may elicit much stronger immunoresponse than does LaSota.

**Table 6b:** Comparison of antibody responses in chicks infected with DHN3 and LaSota.

**Comparison**	**Anti-DHN3**			**Anti-LaSota**			**Anti-DHN3/Anti-LaSota**		
	**7**	**14**	**21**	**7**	**14**	**21**	**7**	**14**	**21 dpi**
rDHN3-mF vs. LaSota	^****^	^****^	^****^						
LaSota vs. rDHN3-mF				^****^	^****^	^****^			
Anti-DHN3 vs. Anti-LaSota							^****^	^****^	^****^

**** *p < 0.0001*.

### Assessment of rDHN3-mF as an Effective Vaccine Candidate

As proved to be lentogenic and highly immnogenic, the protective role of rDHN3-mF against virulent viral challenge was then compared with LaSota vaccine. The inactivated rDHN3-mF vaccine was prepared and used to immunize one-week-old SPF chicks (*n* = 14) followed by challenge with DHN3. Commerical inactivated LaSota vaccine and sterile saline were used as positive and negative immunization controls. Each chick was immunized with a dose of 10^7^EID_50_ vaccine by neck subcutaneous injection and challenged with 10^5^EID_50_ dose of DHN3 by eye and nasal drops at 21 days post-immunization. During this process, five blood samples from each group were randomly collected on 7, 14, and 21dpi for HI. The clinical symptoms and death of the chicks were recorded every day till 7 days post-challenge (dpc). Meanwhile, trachea and cloaca swabs from each group of chickens were collected on 3, 5, and 7 dpc.

After immunization, all chicks appeared normal before challenge with DHN3. Chicks immunized with either inactivated rDHN3-mF or LaSota vaccine did not show any obvious abnormality after challenge; however, chicks immunized with sterile saline started to have syndromes, including drowsiness, loss of appetite, apathetic, and row yellow-greenish dilute feces, on 3 dpc and death from 4 dpc. By 7 dpc, all chicks in this group died. These results confirmed that vaccination with DHN3-mF or LaSota provided effective protection against the DHN3 challenge.

Viral shedding from each vaccinated group was examined by determination of the challenge DHN3 virus from the collected trachea and cloaca swabs by HA assay. As shown in Table 7, immunization with rDHN3-mF vaccine completely blocked DHN3 shedding. However, immunization with LaSota vaccine did not show similar efficacy. Although immunization with LaSota reduced DHN3 shedding, about 20% LaSota-immunized chicks still have viral shedding from cloaca on 7 dpc.

**Table 7:** Viral shedding as detected by HA from trachea and cloaca swabs.

**Group**	**Challenge**	**3**		**5**		**7 dpi**	
		**Trachea**	**Cloaca**	**Trachea**	**Cloaca**	**Trachea**	**Cloaca**
rDHN3-mF	DHN3	9/10	0/10	0/10	0/10	0/10	0/10
LaSota	DHN3	10/10	1/10	4/10	1/10	0/10	2/10
Control	DHN3	10/10	10/10	10/10	10/10	na	Na

*na means not available due to the death of chicks*.

Immunoresponse induced by vaccination was further confirmed by determination of the HI titers and the results were shown in Table 8a. Higher anti-DHN3 HI titers than anti-LaSota were detected in sera collected from chicks immunized with rDHN3-mF. The anti-DHN3 HI titers increased from 4 on 7dpc to 1351 on 21dpc, while the anti-LaSota HI titers only increased from 4 on 7dpc to 56 on 21 dpc (Table 8a). In contrast, in sera collected from chicks immunized with LaSota, the anti-LaSota and anti-DHN3 HI titers increased from 5 on 7dpc to 169 on 21dpc, and from 4 on 7dpc to 56 on 21dpc, respectively (Table 8a).

**Table 8a:** Antibody response against DHN3 and LaSota in the vaccinated chicks.

**Group**	**Anti-DHN3 HI titer**			**Anti-LaSota HI titer**		
	**7**	**14**	**21**	**7**	**14**	**21 dpi**
rDHN3-mF	4 ± 1^ns^	891.44 ± 1.79^****^	1351.17 ± 1.46^****^	3.48 ± 1.37^ns^	73.52 ± 2.14^****^	256 ± 1.64^****^
LaSota	4 ± 1	24.25 ± 3.18	55.72 ± 2.46	5.28 ± 1.46	168.90 ± 1.46	168.90 ± 1.46
Control	1.52 ± 1.46	1.87 ± 1.46	1.32 ± 1.46	2 ± 1	2.30 ± 1.37	2 ± 1.64

*The HI titer is presented as the mean titer ± standard deviation, n = 3*.

Statistical analysis confirmed that vaccination of chicks with rDHN3-mF produced significantly higher antibody response against DHN3, compared to chicks vaccinated with LaSota vaccine on 14 and 21 dpi (Table 8b, rDHN3-mF vs. LaSota). Vaccination of chicks with LaSota also produced significantly higher antibody response against LaSota on 14 and 21 dpi (Table 8b, LaSota vs. rDHN3-mF). However, significantly higher antibody response against DHN3 was induced in rDHN3-mF-vaccinated chicks, compared to anti-LaSota antibody response in chicks vaccinated with LaSota (Table 8b, Anti-DHN3 vs. Anti-LaSota). These results demonstrated that vaccination of chickens with the DHN3-mF vaccine produced much higher antibody titers against the DHN3 challenge than those in the LaSota-immunized chickens.

**Table 8b:** Comparison of antibody responses in the vaccinated chicks.

**Comparison**	**Anti-DHN3**			**Anti-LaSota**			**Anti-DHN3/Anti-LaSota**		
	**7**	**14**	**21**	**7**	**14**	**21**	**7**	**14**	**21 dpi**
rDHN3-mF vs. LaSota	ns	^****^	^****^						
LaSota vs. rDHN3-mF				ns	^****^	^****^			
Anti-DHN3 vs. Anti-LaSota							ns	^****^	^****^

*ns, non-significant*;

**** *p < 0.0001*.

## Discussion

Although ND vaccination has been practiced for more than half a century, a high annual number of outbreaks are repeatedly reported worldwide, demonstrating that current vaccines and vaccination practices are not sufficient to control this disease. First, non-genetically matched vaccines currently being used may lack sufficient efficacy in protection of the vaccinated birds. Non-properly biosecurity practiced in domestic poultry farms may result in the contamination of food and water by sheds of NDV-infected birds. Co-infection with other viruses, such as infectious bronchitis virus, Gallic alphaherpesvirus 1, infectious bursal disease virus etc., may induce immunosuppression thus causing the failure of vaccination. Furthermore, the maternal anti-NDV antibodies also play a role to hinder the immunoresponse induced by vaccination through neutralization of the vaccine antigens.

Genotype-matched vaccines provide a better protection from NDV challenge (32, 33). Traditionally, autogenous vaccines were the first true antigenically matched vaccines used in poultry (34). However, autogenous vaccines generally need many generations of passage *in vivo* (embryo) or *in vitro* (cell line) and it would take time to screen a suitable candidate. In addition, spontaneous mutations may accumulate too many changes over time and may no longer match to the original genotype, leading to the loss of specific protection to the challenge strain. One strategy is to use vector-based recombinant viruses co-expressing NDV major antigenic genes. For example, fowlpox virus (FPV)-based vaccines expressing NDV F or HN protein were able to protect chickens from virulent NDV challenge (35). Two rFPV-ND vaccines are commercially available, but have not been widely used due to the inconvenience in distributing. Another example is herpesvirus-based vaccines, made by inserting the F gene into the thymidine kinase site of MDV genome. Two recombinant rHVTs have been registered and are used internationally. Affected by the presence of maternal antibodies, which neutralize the antigenicity of the vaccine, and the administration limited by cell associated factors, rHVT-based vaccines are also not used widely (36).

In the last few decades, the widely used strategies to rescue the non-fragmented viruses were performed by transfection of the plasmid that expresses the genomic sequence with or without the expression of helper proteins. The most frequently used promoter to drive the genomic cDNA transcription is the powerful T7 polymerase promoter. Other RNA Pol-II promoters also can promote high levels of vRNA expression but are limited by their large size and the transcription termination signal, such as SV40 late poly-adenylation signal. When the T7 system is used, the T7 polymerase must be provided, generally by co-infection with a helper virus, such as fowlpox-T7 or MAV/T7 (37–40). However, removal of helper virus from the rescued final product is a big obstacle (41–44). More importantly, many vaccine registration organizations require rNDV vaccine to be rescued, free from the use of helper virus. In this study, we successfully rescued the recombinant virus rDHN3 from a local isolate of velogenic genotype VII NDV and the attenuated mutant rDHN3-mF, by co-transfection of BHK-21 cells with a plasmid expressing the T7 polymerase, the genomic RNA-expression plasmid and plasmids expressing the axillary protein NP, P, and L.

To compare the rescue efficiency of this helper virus-free, plasmid-based system with a helper virus-based system, the vaccinia/T7 recombinant virus (V/T7) was used as a helper virus in parallel during the initial rescues of the combinant NDV. In the first few repeated experiments, recombinant NDV was rescued from both systems with the same efficiency, except that typical CPE appeared ~1–2 days earlier when the V/T7 helper virus was used (unpublished observations). It suggests that, by using the full-length NDV cDNA as a template, a slightly less and delayed, but sufficient amount of the negative stranded viral genomic RNA would be transcribed in cells transfected with the T7 Polymerase-coding plasmid, consequently resulting in efficient assembly and rescue of the infectious NDV with the co-expressed viral NP, P and L proteins. As removal of the helper V/T7 from the rescued recombinant NDV would be an obstacle for further vaccine development and due to the clear advantage and convenience of the plasmid-based system, we decided not to use the helper virus in the following rescue experiments. This helper virus-free, plasmid-based approach proved to be simple, fast, reliable and efficient, and would be readily adoptable to other negative-stranded RNA viruses.

Extensive studies have demonstrated that NDV virulence is correlated with the cleavage of F protein into F1 and F2 by cellular proteases (30, 45, 46). The monobasic amino acid motif at the cleavage site, 112GR/K-Q-G-R↓L117, found in all lentogenic groups, are cleaved extracellularly by trypsin-like proteases in the respiratory and intestinal tracts, leading to a narrowed cell tropism and mild disease. The multi-basic amino acid motif at the cleavage site, 112R/G/K-R-Q/K-K/R-R-F117, found in mesogenic and velogenic groups, are cleaved intracellularly by the ubiquitous furin-like proteases (29–31), resulting in a wider cell tropism and severe systemic infection. Based on sequence and phylogenetic analyses, the locally isolated DHN3 was identified as a VII genotype and velogenic strain. In addition to the presence of the multi-basic amino acid motif at the cleavage site, its virulence was further confirmed by the pathogenic tests including the EID_50_, MDT and the clinical symptoms in DHN3-infected chicks. Attenuation of this virus was achieved by replacing the 112–117 residues with the corresponding sequence from the lentogenic strain LaSota. This is consistent with other published studies, showing that converting the cleavage site from the lentogenic strain to that of velogenic strain increased viral virulence significantly (40, 47, 48). However, the recombinant NDV strain NDFLtag, a derivative of LaSota strain containing a velogenic cleavage site, showed only a minor increase of virulence (49), suggesting that more factors may contribute to the virulence and clinical symptoms of NDV.

Whether a live or an inactivated ND vaccine was used, the level of virulent challenge virus shed from birds immunized with a homologous vaccine was lower than that immunized with a heterologous vaccine (12, 50). In consistence, our results demonstrated that the level of specific antibody response against DHN3 is higher than that of anti-LaSota response in the rDHN3-mF-immunized chicks. This would confer a stronger protection against DHN3 challenge to the immunized chicks, and lead to more efficient control of disease and reduced viral shedding. Chicks immunized with the DHN3-mF vaccine also showed much earlier viral clearance, compared to those immunized with the LaSota vaccine.

Infection of chicken embryos with rDHN3-mF would produce more HA antigen, as shown by the HA test. Although the viral titers determined by TCID_50_ were similar among rDHN3, rDHN3-mF and DHN3 stocks, the HA value in the allantoic fluid collected from rDHN3-mF-infected embryos was consistently higher (9log2) than that from rDHN3- and DHN3-infected embryos (8log2). The underlying mechanism is yet to be understood, but it may be related to the stability of the F protein. A recent study showed that mutation of the velogenic strain rSG10 F gene increased the viral thermostability but reduced the viral pathogenicity, neuraminidase, hemadsorption and hemolytic activities as well as the fusogenic capacity, demonstrating that the NDV F gene is associated with the viral thermostability and antigenicity (51). Nevertheless, the higher yield of the HA protein produced in chicken embryos infected with this mutant virus would be beneficial when a vaccine is developed based on this virus. Compared with the conventional ways in developing a vaccine strain from a field isolate, including inoculation of chicken embryonated eggs, this rapid protocol would have economic advantages.

In summary, this study provided a reliable system for the rapid generation of infectious cDNA clone by *in vitro* recombination method and efficient rescue of the recombinant virus with a T7 polymerase expressing plasmid. The attenuated recombinant virus rDHN3-mF created by change of the furin-cleavage side motif (112–117) from velogenic type to lentogenic type drastically reduced viral virulence. As a genotype-matched vaccine candidate, this mutant virus can be further developed into a vaccine product with the advantages of stimulating specific antibody responses, preventing viral shedding, and protecting birds against the virulent VII type NDV infection.

## Data Availability Statement

The datasets presented in this study can be found in online repositories. The names of the repository/repositories and accession number(s) can be found here: https://www.ncbi.nlm.nih.gov/genbank/, MT447874.

## Ethics Statement

The animal study was reviewed and approved by Animal Welfare and Ethical Censor Committee at South China Agricultural University.

## Author Contributions

RC and DL designed and organized the study. NW did most of the experimental work. QL, JY, QD, and LW did parts of the experimental work. MH and NW analyzed the data. TF worked on the figures. MH and DL wrote the manuscript. All authors contributed to the article and approved the submitted version.

## Conflict of Interest

MH, QL, JY, LW, and RC were employed by the company Zhaoqing Institute of Biotechnology Co., Ltd., China. The remaining authors declare that the research was conducted in the absence of any commercial or financial relationships that could be construed as a potential conflict of interest.
